# Supplementary material for: Conditional chemoconnectomics (cCCTomics) as a strategy for efficient and conditional targeting of chemical transmission
Source: eLife. 2024 Apr 30;12:RP91927. doi: 10.7554/eLife.91927 (PMC11060718; doi:10.7554/eLife.91927)
Supplement: Figure 3—source data 3. [file elife-91927-fig3-data3.zip › Figure 3 source data3 part 2.zip!/Figure 3-Source data 3-part2/1062-13-CRISPResso_Report_jt28fhr9.zip!/CRISPResso_on_1062_13/3b.gcaggaaatgagctacctac.Insertion_deletion_substitutions_size_hist.pdf]

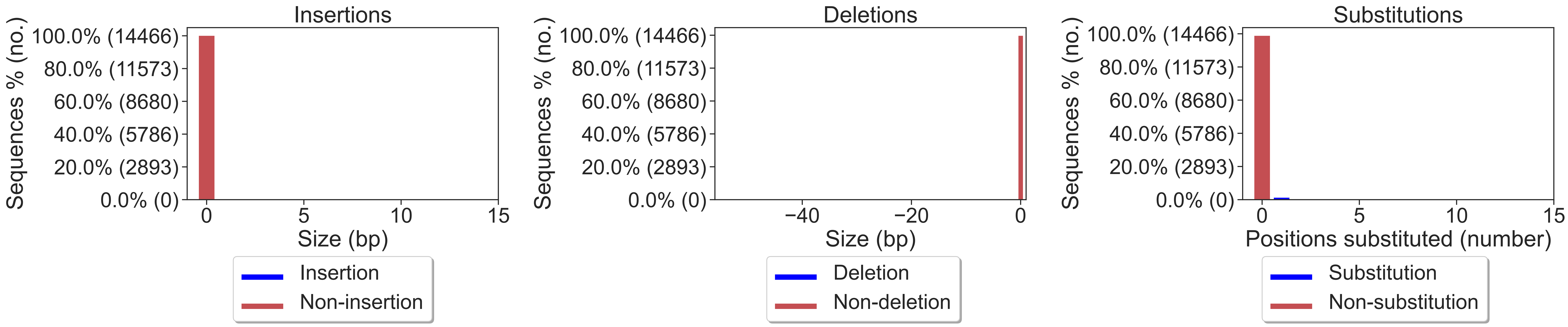
Insertions
Deletions
Substitutions
Sequences % (no.)
100.0% (14466)
80.0% (11573)
60.0% (8680)
40.0% (5786)
20.0% (2893)
0.0% (0)
0
5
10
15
−40
−20
Size (bp)
Positions substituted (number)
Insertion
Non-insertion
Deletion
Non-deletion
Substitution
Non-substitution
